# Can electronic-cigarette vaping cause cancer?

**DOI:** 10.46439/cancerbiology.2.027

**Published:** 2021

**Authors:** Moon-shong Tang, Yen-Len Tang

**Affiliations:** 1 Professor of Environmental Medicine, Pathology and Medicine, New York University School of Medicine, New York, NY 10016, USA; 2 Pediatrician, Berkeley, CA, USA

The relative safety of E-cigarette (E-cig) has been an emerging topic in the public domain as well as the medical and scientific communities as vaping associated health problems arose. While there were significant amounts of intelligent discussions and opinions on the benefits and deleterious effects of E-cig vaping, there is a lack of solid evidence of the fundamental biochemical and biological effects of E-cig aerosol and nicotine.

Four years ago, in response to the popularity of E-cig vaping—particularly among young adults— and the message of E-cig vaping as a safer alternative to tobacco smoke being permeated in the scientific community and media, we started to revisit the question of the biochemical and biological effects of nicotine. Our focus has been on whether or not nicotine is carcinogenic.

It should be noted that within the scientific community it is generally believed that while nicotine causes human addiction and other neurological disorders, it is not a carcinogen in humans and in rodents. Most cited evidence that leads to this conclusion is based on a publication by Waldum et al. [1,2] that show that rats exposed to stream-air-vaporized nicotine via inhalation for two years showed no sign of tumor formation, including lung tumors. On the other hand, different types of tumors including leiomyosarcoma were observed in animals treated with nicotine via drinking water and subcutaneous injection [3]. All these results were criticized for lacking necessary bioassays in addition to very small sample sizes (22 exposed versus 6 control) and other experimental shortcomings. As a result, they were deemed to be inadequate evidence for an association between nicotine exposure and its effect on carcinogenesis [4]. Despite these inconclusive results, the prevailing thinking remains that nicotine is non-carcinogenic [2]. Is this collective wisdom, or a myth? Where does the truth reside?

It is common practice that during tobacco curing, sodium nitrites are added as a preservative to prevent microbe growth. As a result, a chemical reaction called nitrosation takes place between nicotine and nitrite transforming it into nitrosamines. This transformation can also occur during tobacco burning [5]. Many of these nitrosamines, NNK (4-methylnitrosamino)-1-(3-pyridyl)-1-butanone) and NNN (*N*-Nitrosonornicotine) in particular, are potently carcinogenic in both humans and animal models; they can cause lung, oral cavity esophageal tumors in animal models [5]. So, in reality nicotine is ONLY ONE STEP away becoming potent human and animal carcinogens in many conditions. In essence the question of whether or not nicotine, and for practical matter E-cig, is carcinogenic is the same as the question of whether nicotine nitrosation can occur in humans and animals. The concern for nitrosamine exposure has already led to FDA recalls and current focus on common medications such as ranitidine and angiotensin receptor blockers with levels of nitrosamine contamination due to their classification as a probable carcinogen [6].

## Experiments to Demonstrate Nicotine and E-cigarette Aerosol Effects

With this backdrop, we first determined the biochemical and biological effects of nicotine and the nitrosamine NNK in cultured human lung and bladder epithelial cells. It is well established that inside cells, NNK is readily further metabolized and degraded into products which can cause DNA damage and that NNK carcinogenicity has been attributed to the mutations induced by the DNA damage [5]. In our experiments, we measured the DNA damage induced by nicotine in cultured human cells. We also measured DNA damage induced by NNK in the same human cells in parallel. More importantly, we identified and measured the level of DNA damage induced in different organs of mice exposed to E-cigarette aerosol [7].

## Results and Evidence!

To make a long story short, we have two important findings: First, we found that nicotine and NNK induce not only the same type of DNA damage, but also the same inhibitory effect on DNA repair mechanisms in human lung and bladder epithelial cells [7]. These two compounds also make these human cells more susceptible to mutations and tumorigenic transformation. Since mutations and tumorigenic cell transformation are two preludes of tumorigenesis, these results indicate that in human cells nicotine can be converted to nitrosamines and further metabolized into DNA damaging products which exert carcinogenic effects. Second, we found that the E-cig aerosol induces the same type of DNA damage in lung, heart, and bladder tissue of E-cig exposed mice. E-cig aerosol also causes inhibition of DNA repair and reduction of DNA repair proteins in the lungs of mice. These results strongly suggest that *in vivo* nicotine can be nitrosated into NNK and other nitrosamines and further metabolized into DNA damaging products which can cause carcinogenic effects [7].

Naturally, the next step we undertook is to determine whether E-cig aerosol can cause cancer in mice. To do this, we exposed mice with commercially available E-cig juice via vaping for 54 weeks, an amount of exposure roughly equivalent to 3 to 6 years of vaping in humans. We found that in E-cig exposed mice, 9 out of 40 (22.5%) developed lung adenocarcinoma, and 23 out of 40 (57.5%) developed bladder urothelial hyperplasia, a pre-cancer stage. In contrast, in control mice, only one out of 38 developed lung cancer and one developed bladder urothelial hyperplasia. Based on these results we concluded that nicotine can induce tumorigenic effects in cultured human cells and that E-cig aerosol can induce the same tumorigenic effects in mice, and moreover induces lung cancer and precancer pathological changes in bladder in mice [8].

## Will E-cigarette Aerosols Cause Human Cancer?

Of course, our result that E-cig aerosol causes cancer in mice does not mean that E-cig aerosol can cause cancer in humans, at least not yet. In general, it takes more than two decades for a life-time tobacco smoker to develop cancer. E-cigs became popular just over eight years ago. If tobacco smoke carcinogenesis is a paradigm for E-cig carcinogenesis in humans then we may not see the emergence of E-cig aerosol associated human cancers for a decade to come. However, one thing we have to keep in mind is that all the E-cig effects – DNA damage, DNA repair inhibition, lung cancer and bladder precancer pathogenesis – in mice can be attributed to NNK and NNN, which are proven potent carcinogens in humans and mice. So, based on these results, it is sensible to conclude that there is a high probability of E-cig aerosol being a human carcinogen. Therefore, there is no reasonable basis for E-cig users to assume that E-cig aerosol does not cause cancer, and it is likely a dangerous step.

Unfortunately, the E-cig industry is conducting a gamble on the perception of E-cig safety in the human population and simultaneously making a lucrative profit. Currently more than 11 million adults, among them 4 million young adults, are using E-cig in the US alone [9]. This is tantamount to a large human experiment on the effects of E-cig vapors. Although no human cancer associated with E-cig vaping has been reported so far, hundreds of lung illnesses and scores of deaths are attributed to E-cig vaping [10–15]. Since nicotine can damage genetic materials, it certainly can damage cells no matter how small the effect is. It is foreseeable that frequent E-cig vaping will accumulate more “damage” in the organs, eventually reaching a threshold which can manifest as human pathology. In regards to E-cig related cancer, it will likely take two decades or more to develop. Therefore, the jury will come out in a decade or so of whether E-cig is carcinogenic to humans. Our findings in mice, that verdict has already become clear.
